# Occupational therapists' experiences in Australia's primary mental health funding scheme: Navigating complexity in a constrained system

**DOI:** 10.1111/1440-1630.70104

**Published:** 2026-07-01

**Authors:** Arabella Hely, Claire Pearce

**Affiliations:** ^1^ Faculty of Health University of Canberra Bruce Australian Capital Territory Australia

**Keywords:** advocacy, health policy, Medicare, mental health services, occupational therapy, primary health care, professional role, qualitative research, recovery‐oriented care, systemic barriers

## Abstract

**Introduction:**

Occupational therapists in the Australian context provide significant value to mental health, including through Better Access, a Medicare initiative that gives individuals access to up to 10 sessions with a psychologist, social worker, or occupational therapist following a referral from a medical practitioner. However, the experiences of occupational therapists working under the initiative are not widely understood, with no recent occupational therapy‐specific research. This study aims to explore the experiences of occupational therapists working under Better Access, their distinct role, and how the system shapes the care clients receive.

**Methods:**

The study used a qualitative, inductive approach. Thirteen occupational therapists who work with people funded by Better Access participated in 30‐minute, semi‐structured interviews. Data were analysed via thematic analysis.

**Consumer and Community Involvement:**

The lead researcher for this study has lived experience of mental illness and Better Access, but further consumer and community involvement was not undertaken.

**Findings:**

Analysis generated themes highlighting the complexity of occupational therapy practice within a psychology‐centric system. Participants described how occupational therapists drew on specialised skills to support clients with complex needs, yet worked within structural constraints such as restrictive practice rules, session caps, and limited remuneration. Themes also reflected the undervaluation of occupational therapy and the need for ongoing advocacy to ensure equitable access to services, appropriate funding, and recognition of the profession's contribution.

**Conclusion:**

The design of Better Access restricts the ability of occupational therapists to provide meaningful and sustainable occupational therapy services. Findings provide further evidence that longstanding issues identified in early research have persisted and that the burden occupational therapists face of consistently advocating for the profession and their clients is symptomatic of broader structural inequities. Findings highlight the need for systemic reform.

## PLAIN LANGUAGE SUMMARY

This study looked at the experiences of occupational therapists working under the Better Access initiative. Better Access is a program that helps people with mental health concerns see professionals like psychologists, social workers, and occupational therapists. Whereas there is a lot of research about psychology in Better Access, there is very little about occupational therapy. We wanted to understand what occupational therapists do in this program and how the system affects the care people receive.

We interviewed 13 occupational therapists about their work under Better Access. They told us that occupational therapists are skilled at helping people with complex needs. However, Better Access is not designed for this type of care. The program focusses more on psychology, which limits how occupational therapists can use their own approaches. Many referrers do not know that occupational therapy is an option, that the program offers only 10 sessions, or that the payment for occupational therapy is much lower than for psychology. These factors make it hard for occupational therapists to provide recovery‐focussed, practical support and to keep their practices going.

These issues mean that clients often cannot get the occupational therapy care they need. Changes are needed to make the system fairer and more effective. These could include raising awareness of occupational therapy, allowing greater flexibility in care, and increasing payments to align with psychology. Our study was small and did not include doctors or clients, which is a limitation. Still, it helps show the challenges occupational therapists face and why Better Access needs reform.

**Key Points for Occupational Therapy**

- Occupational therapists are essential for meeting the complex mental health needs of consumers.
- The Australian primary health funding scheme, Better Access for Mental Health, restricts occupational therapy practice and limits client access to occupational therapy.
- Systemic reform is needed to address the structural barriers to clients accessing occupational therapy.

## 1 INTRODUCTION

Primary health, allied health, and mental health services in Australia are funded through the Better Access initiative, established in 2006 to improve access to treatment for individuals with mild to moderate mental health conditions (Australian Bureau of Statistics, 2020–2022). Access to the program requires a referral and a mental health treatment plan from a general practitioner (GP), psychiatrist, or paediatrician and provides up to 10 subsidised sessions with an eligible psychologist, social worker, or occupational therapist. To practise under Better Access, occupational therapists must obtain endorsement by Occupational Therapy Australia (Occupational Therapy Australia, 2025). Under the initiative, they can provide approved focussed psychological strategies, including psychoeducation, cognitive behavioural therapy, skills training, eye movement desensitisation and reprocessing therapy (EMDR), and narrative therapy. Occupational therapists may experience role ambiguity because these focussed psychological strategies are generic and non‐discipline specific (Hely & Pearce, 2025).

Data collected within the first 12 months of the Better Access scheme, when just 143 occupational therapists were practising under the initiative, found that occupational therapists experienced persistent challenges related to relationships with referrers, professional identity, and dissatisfaction with their remuneration, especially in comparison with psychologists (Hitch, 2009). In another study exploring the impact of occupational therapy under the initiative, Kohn et al. (2012) found that occupational therapy services reduced psychological distress. Both Hitch (2009) and Kohn et al. (2012) highlighted the under‐representation of occupational therapists in Better Access research and called for further investigation into the profession's role and contributions within the scheme.

There are broader challenges with the Better Access scheme. Limitations include inequitable access for rural and low‐income populations (Meadows et al., 2015), insufficient session numbers for people with complex or chronic mental health needs, and the scheme's tendency to privilege short‐term, individualised therapy over multidisciplinary or community‐based approaches (Papadopoulos & Maylea, 2020). An independent evaluation of Better Access (Pirkis et al., 2022) found that although effective for short‐term treatment, the design of the initiative led to limited opportunities to address the long‐term needs of clients. The report recommended expanding the mental health workforce, increasing affordability, improving collaboration between referrers and providers, funding family/carer consultation items to increase holistic care, and allowing for an additional 10 sessions for individuals with complex mental health concerns (Pirkis et al., 2022).

In Australia, Better Access operates as one component of a broader, multi‐layered mental health funding system. State and territory governments fund public mental health services, including acute, community, and crisis care. At the federal level, the National Disability Insurance Scheme (NDIS) funds long‐term, capacity‐building supports for people with permanent and significant psycho‐social disability. Unlike Better Access, which provides short‐term, treatment‐focussed interventions (Pirkis et al., 2022), the NDIS enables ongoing occupational therapy aimed at functional recovery, community participation, and daily living. Understanding these parallel systems is essential, as occupational therapists frequently navigate the boundaries between federal and state schemes, each with different eligibility criteria, scopes of practice, and implications for access to occupational therapy.

Earlier studies provide useful insights, but there has been no recent occupational therapy‐specific research to reflect the current policy context of Better Access, including the impact of other schemes such as the NDIS. This limits understanding of how occupational therapists working within primary health care navigate systemic barriers, advocate for their role, and deliver mental health services, as well as how clients access (or fail to access) occupational therapy. Following the independent evaluation of Better Access (Pirkis et al., 2022), re‐examining the contemporary contribution of occupational therapy is both timely and necessary.

This study addresses an evidence gap by providing contemporary insights into how occupational therapists experience their role within Better Access.

The research aims to

- explore the distinct role of occupational therapy within Better Access;
- explore how occupational therapy is understood and recognised within the Better Access system; and
- examine how the structure and funding model of Better Access shape the care clients receive.

## 2 METHODS

### 2.1 Research design

This research was designed and conducted within a critical‐interpretivist paradigm. This recognises that individuals' perspectives are shaped by their lived experiences, social interactions, and the broader structural and policy contexts (Stanley & Nayar, 2024). A qualitative, inductive design was used to explore occupational therapists' perspectives of working under the Better Access initiative. An inductive approach was appropriate because the area remains under‐examined and created space for participants' meanings, interpretations, and contextual realities to shape the analysis rather than imposing predefined categories. This aligns with a constructivist perspective, which assumes that knowledge is co‐constructed through interaction and that participants' accounts reflect both individual experience and the broader social, professional, and policy environments in which they practise (Charmaz, 2014).

Data analysis was undertaken using thematic analysis (Braun & Clarke, 2006) supported by reflexivity. This approach recognises the researcher as an active meaning‐maker, not a neutral observer, and positions themes as analytic outputs generated through deep engagement with the data rather than as entities waiting to be discovered (Braun & Clarke, 2019). Reflexivity was embedded throughout the process, including ongoing consideration of how the researchers' professional and personal backgrounds and assumptions shaped decisions about coding and theme development. The primary researcher (Author 1) maintained a reflexive journal, and regular discussions were held between Author 1 and Author 2 as analysis progressed. This reflexive stance supported transparency and ensured that the analysis remained grounded in participants' accounts while being attentive to the structural and systemic contexts influencing their experiences.

### 2.2 Positionality statement

Author 1 (A. H.) is an occupational therapist who undertook this research as part of their Bachelor of Occupational Therapy Honours. They have lived experience of mental illness and the Australian mental health care system, including as a consumer in the Better Access initiative. Author 1's knowledge of and beliefs surrounding mental health care are influenced by these experiences. Although these experiences have contributed to comprehensive insights into the Australian mental health care system, it is acknowledged that they may have potential for bias. To mitigate this, throughout the research process, Author 1 engaged in regular discussions with research supervisor Author 2 alongside regular self‐reflection and reflexive journaling.

Author 2 (C. P.) is an Australian‐born occupational therapist who works as an academic. They were the supervisor of Author 1's Honours research. Author 1 has worked within mental health systems in clinical and management roles. They acknowledge that their profession and position as a white cisgender woman place them in a position of privilege.

### 2.3 Participants

Purposive sampling was utilised once ethics approval was granted from the University of Canberra (Project ID: 13377). Purposive sampling deliberately selects participants who can provide relevant and useful insights (Palinkas et al., 2015). The study was shared on LinkedIn and professional networks before being published in Occupational Therapy Australia's news bulletin. By publishing in Occupational Therapy Australia rather than limiting to the researchers' professional networks and geographic location, a more diverse selection of participants was available. Snowball sampling was also used when participants circulated the recruitment flyer within their professional networks, further expanding the reach (Noy, 2008).

Participants were required to be occupational therapists currently practising under the Better Access initiative. As is consistent with reflexive thematic analysis, the study did not seek data saturation, which assumes that meaning is finite and discoverable. Instead, participant recruitment continued until the dataset was sufficiently rich and diverse to address the research aims (Braun & Clarke, 2021; Malterud et al., 2016). Thirteen participants from diverse practice settings and locations across Australia were recruited, whose varied perspectives enhanced the depth and richness of the data (Palinkas et al., 2015). Information related to the participants' work, geographical setting, or clinical experience practice has not been included to maintain anonymity.

### 2.4 Data collection

Semi‐structured interviews were conducted via Microsoft Teams or telephone after consent had been obtained. The interviews focussed on exploring participants' experiences of working under the Better Access initiative, with open‐ended questions enabling participants to provide candid, rich responses (Jamshed, 2014). The interview guide, designed to prompt reflection while remaining adaptable to each participant's narrative, is provided in Appendix S1. Interviews were audio and video recorded and lasted an average of 30 minutes, ranging from 25 to 45 minutes.

### 2.5 Data analysis

Interviews were recorded and transcribed verbatim via Microsoft Teams, then checked against recordings for accuracy, and edited by Author 1. Verbatim transcription helps preserve participants' voices and supports nuanced interpretation of language, tone, and meaning, reducing researcher bias in the early analytical stages (Davidson, 2009). Personal information and identifiable data were removed, and participants were assigned a participant identifier. Reflexive memos were maintained throughout to document analytic decisions and researcher positioning. Initial coding was conducted by Author 1, with regular collaborative discussions with Author 2 to enhance rigour and trustworthiness (Nowell et al., 2017). The full data analysis process follows Braun and Clarke's (2006) six‐step thematic analysis, as summarised in Table 1.

**TABLE 1:** Data analysis process using Braun and Clarke's thematic analysis (2006).

Familiarisation with data	Author 1 immersed themselves in the data by listening to recordings of interviews multiple times, rereading transcripts, and recording early impressions in a reflexive journal.
Generating initial codes	Data were coded manually in Microsoft Word (Clarke et al., 2021) following inductive reasoning, with codes tied to participants' words rather than pre‐existing theory. Codes were discussed regularly with Author 2.
Constructing initial themes	Codes were further analysed with coded segments reviewed and grouped into broader patterns. Themes were constructed based on how groups fit with the research questions.
Reviewing and refining	Developed themes were reviewed collectively and against the dataset to ensure suitability and were then discussed and refined by Author 1 and Author 2.
Defining and naming themes	Themes were further refined to identify central concepts, with clear names and descriptors developed that reflect meaning and relevance to research aims.
Producing the report	Themes were written up with quotes to represent participant voices.

## 3 FINDINGS

Analysis of the data identified four themes and supporting subthemes, as summarised in Table 2.

**TABLE 2:** Themes and subthemes.

Theme	Subtheme
Occupational therapy's value in addressing complexity	Working in an initiative not designed for complexity
	Occupational therapy as holistic, broad, and practical
Operating within a psychology‐centric system	Lack of awareness and recognition
	Fitting into focussed psychological strategies
Financial and structural constraints	Rebates and financial sustainability
	Limitation of 10 sessions
The necessary role of the occupational therapist advocate	Ensuring clients receive the best possible care
	Promoting occupational therapy

**FIGURE 1:**
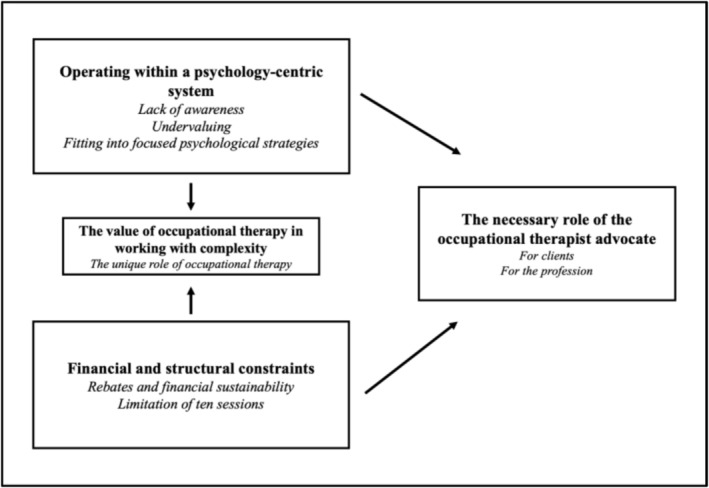
Tensions within Better Access for occupational therapy.

Together, as presented in Figure 1, these findings illustrate the tension between the structure of Better Access and the principles of occupational therapy.

### 3.1 Occupational therapy's value in addressing complexity

Participants described their role as particularly well suited to supporting clients with complex needs. This complexity was considered misaligned with the assumptions underpinning the Better Access initiative, which participants felt privileged short‐term and more straightforward presentations.

People are complex … I think the idea the government has that just somebody has anxiety or just depression and they're just worried. Well, the reality is that a lot of people that we see have really complex … there's been an underrepresentation of the impact of trauma in people's lives … typical mental health diagnostic groups don't as easily represent the level of complexity and trauma that people are living. (OT2)

Participants noted that the initiative does not adequately address their clients' needs, describing working with individuals experiencing chronic and complex mental health conditions, often compounded by factors such as family violence, unstable housing, and financial concerns. These complexities required sustainable and adaptable care, which participants identified as a particular strength of occupational therapy.

I'm really passionate about OT having that skill set to understand all of the realms across mental health … not just the social determinants of health or the psychological space, but we have that bridge across physical, social and psychological as well. (OT9)

Participants also described their broad and diverse skill sets. These were often complemented by additional training and professional development in areas such as EMDR and specific practice areas, including perinatal mental health, eating disorders, and neurodiversity‐affirming care.

I think we've got quite a broad set, with our sensory, our ADL [activities of daily living], our mental health training. …. We've got a broad training which helps. (OT12)

The broad nature and overarching values of occupational therapy allowed them to translate psychological interventions into everyday life. Participants framed this as a key strength of occupational therapy in promoting functional outcomes and long‐term recovery.

I'm here to try and help you actually put into practice a lot of what you've worked through …. Let's actually try to see how that core belief now looks like in practice, how do we actually do that stuff. (OT1)

Collectively, participants described themselves as problem‐solvers. They noted skills in navigating ambiguity and tailoring supports that worked for the individual in the context of their everyday life.

I think we're quite good at the complex problems with complex physical and mental health disabilities … trying to help them improve their functioning. (OT8)

Those core OT skills … the collaboration, the client focus, the neurodiverse affirming, trauma‐informed care, activity analysis. All of those are to make us really good at things … being able to sift through complexity, pick a focus, and achieve something that works for the person. (OT3)

Overall, participants described occupational therapy as practical, client centred, and holistic and reflected on the benefits of this in working with clients experiencing complexity.

### 3.2 Operating within a psychology‐centric system

Occupational therapists described the challenges of working within a psychology‐dominated framework under the Better Access initiative, which was seen as often failing to reflect the scope and distinct contributions of occupational therapy.

Participants consistently reported a lack of understanding and recognition of occupational therapy's role among referrers, particularly GPs, and several expressed frustrations that occupational therapy was frequently overlooked or misunderstood in the referral process.

I think there's just a lack of knowledge and yeah, probably I'm going to say it, but willingness to kind of be open to that as well. (OT11)

I think initially most GPs wouldn't have a clue that mental health OTs are involved or can deliver that service. (OT6)

Psychologists were frequently described as the default mental health professionals, with ingrained assumptions by other health professionals that they were more qualified to work with complex cases.

I guess it's just, so that idea that psychologists are the therapists, and that's what they've always been. That's what they're known as. (OT5)

The GPs … will sort of say to clients like, ‘are you sure you shouldn't be seeing a psychologist?’ (OT3)

Participants described struggling to fit their unique scope of practice as occupational therapists within the constraints of the Better Access model, particularly the requirement to practise under focussed psychological strategies. Although they had training in psychological interventions such as cognitive and dialectical behavioural therapy, counselling, and acceptance and commitment therapy, many occupational therapists found the narrow framing of the initiative limiting and at times misaligned with client needs.

At the end of the day we have to be doing these psychological‐focused strategies, but we're not psychologists, so we're very restricted in that sense … we're limited in our capacity to do some more OT stuff. (OT11)

Despite these limitations, many occupational therapists described ways in which they adapted traditional psychological therapies through an occupational therapy lens, bringing a focus on function, participation, and meaningful occupation into their work.

Well, there's what's allowed under the scheme, right? …. We're expected to adhere to this quite short list of evidence‐based treatments, but I really think you have to bring in your occupational therapy skills on top of that. (OT8)

Several participants expressed concern over role blurring and the pressure to adopt a psychologist‐like identity to conform to the initiative's requirements.

I do sometimes worry like if I'm doing some sensory work in the Better Access space … even though that can be under anxiety management, it's bordering on not being specific to psychological therapy. … I really have to put on like a psychologist hat when I'm doing that work, and I'm not a psychologist. (OT11)

Under the initiative, occupational therapists demonstrated adaptability in delivering care. However, they also indicated ongoing frustration with the position psychology occupies as the dominant mode of mental health practice, often at the expense of recognising the distinct value of occupational therapy.

### 3.3 Financial and structural constraints

#### 3.3.1 Rebates and financial sustainability

Occupational therapists consistently described the challenges of working within the financial and structural constraints of the Better Access scheme. A key concern was the limited Medicare rebate received for providing Focused Psychological Strategies, particularly when compared to clinical psychologists.

I have no issue with psychologists getting a higher rebate for doing something that an OT can't do. But if we're doing exactly the same intervention … then we should be getting the same rebate. (OT6)

Participants, especially those who bulk‐billed clients, also expressed concern about the financial viability of their practices.

You can't live off the rebate like bulk billing … I don't think we're properly reimbursed for our skill level. (OT11)

This resulted in ethical and practical tensions between the desire to provide accessible services and the need to maintain a sustainable business.

It's a tricky space for an OT to be in because you want to help everyone, but you've also got to, you know, keep a roof over your head as well. (OT13)

In response to these constraints, occupational therapists described the need to diversify their funding sources. Many worked across multiple schemes, including the NDIS, chronic disease management plans, eating disorder care plans, workers' compensation schemes, and private billing.

That's why I actually cover so many different funds, because you're trying to have enough breadth to actually create an income and run a business. (OT7)

#### 3.3.2 Limitation of 10 sessions

The initiative's limit of 10 subsidised sessions per year was consistently cited as a significant constraint, especially when working with individuals with complex needs. Participants explained that these limitations reduced their capacity to provide ongoing, holistic occupational therapy for these clients.

What would help is extending the number of sessions so that you could be given time for that rehabilitation component … when they're recovering, they're more open to addressing their functioning … engaging in leisure, finding meaningful activities, adjusting to role transition. (OT8)

In many cases, participants noted that the need to stretch sessions over an entire year or prioritise crisis management over long‐term goals led to fragmented or ‘patchwork’ care, limiting the depth and impact of the intervention.

Participants also reflected on the tension between individual and systemic approaches to care. Many viewed occupational therapy as holistic and requiring a whole‐of‐family approach, especially when working with children and young people. However, they found the limited number of sessions directly impeding this work.

It's important to involve family members … and that kind of eats into the number of actual individual therapy sessions. So it kind of makes you lean towards the individual work. (OT9)

These financial and structural limitations were described as deeply misaligned with both the nature of occupational therapy and the needs of the client populations that occupational therapists support. These limitations directly shaped how occupational therapists delivered care, who could access it, and what outcomes were possible.

### 3.4 The necessary role of the occupational therapist advocate

Occupational therapists described how operating within a psychology‐centric system embedded with significant financial and structural constraints often necessitated taking an additional role—that of the advocate. This advocacy was described as integral to enabling clients to access appropriate and continued care, while also promoting the professional contribution of occupational therapy within a framework that often failed to recognise its scope or distinct value.

#### 3.4.1 Ensuring clients receive the best possible care

Occupational therapists described needing to advocate for their clients to ensure they could access and receive care. Often seeing clients with complex, long‐term needs, the limitation of 10 sessions requires occupational therapists to be creative in their approach, finding workarounds such as utilising online therapy modules. Participants working with children also often suggested and assisted caregivers to obtain their own mental health care plans for use with the occupational therapist.

A lot of people that come to see seek help have more complex problems than fits a sort of a 10 session model with a beginning and an end. We, you find these sort of workarounds there's, there's, you know what the rules are, then there's what the reality of clinical practice and what your clients actually need. (OT8)

Occupational therapists working in low‐income areas described the unique challenges of ensuring care remains accessible to their most at‐risk clients. For many, this meant bulk billing, despite the financial implications.

I work in an area that's low socioeconomic. People are barely making ends meet and not eating properly. You know, who am I to ask them to pay me more? … none of the local psychologists are covering that need. (OT7)

Within these constraints, occupational therapists described continuing to advocate for clients to receive adequate care, whether through supporting clients to access the NDIS, My Aged Care, or funding from other sources such as domestic violence or defence support funding. This additional role can take up time from the 10 sessions available or will be an additional workload for occupational therapists on top of their clinical and regular administrative work.

You do work across many funding models and often that's not a choice, it's because that's how I can remain servicing people in a regional area who are vulnerable … I'm writing letters back to GPs and suggesting, like, this person might need extra referrals here or there, everywhere. (OT1)

Because Better Access is limited … if a child does seem to be eligible for NDIS, it's quite common that I will try to put forward some evidence to get them onto NDIS … my aim is that the child is not left in the lurch at the end. (OT12)

Participants spoke about the difficulty clients often faced in advocating for themselves, especially those with severe mental health concerns, histories of trauma, or significant social disadvantage. In response, occupational therapists felt they naturally stepped into the role of advocate.

These people don't have it in them to fight for what they need … and I think that's one of the things we OTs fall into a lot, is into the advocacy role. We often end up advocating for folk … to get them plugged into My Aged Care …. There's a lot of advocacy work, and that's kind of additional to counselling. (OT7)

#### 3.4.2 Promoting occupational therapy

As a result of working within a psychology‐centric system with significant structural limitations, occupational therapists also often needed to advocate for themselves and the profession to ensure that clients were being referred to the appropriate service. Participants described the importance of relationship building when working with referrers and other health professionals.

It's building up a reputation to see, to get the referral‐ referrals through the door. It doesn't happen automatically. (OT4)

Occupational therapists also described how they were required to engage in extensive self‐promotion and education to GPs, feeling that ‘it all feels a bit sales‐y’ (OT5) and that they needed to market themselves ‘like our lives depend on it’ (OT5). Often, this was in the form of additional letters informing GPs of their service offering and how occupational therapy could benefit clients with mental health concerns.

I do have a letter to send to a GP to and with all the websites, so I have to keep that up to date … ‘yes, OTs are in this scheme’. (OT10)

Although these efforts were often driven by individual occupational therapists, many described the need for it to occur at a systemic level, with calls for greater recognition from peak bodies and government agencies.

Everybody in government, anyone who's a spokesperson for mental health anywhere, need to be receiving communication from OT Australia that we are, we are one of the three professions that deliver this program. There is not just one, there are three. (OT6)

## 4 DISCUSSION

This research explored occupational therapists' experiences working within the Better Access initiative. It aimed to examine how the initiative shapes the role of occupational therapists and the care clients receive. Analysis identified that the value of occupational therapy under Better Access lies in the ability to work with clients with complexity. However, occupational therapists face substantial pressures from practising under a psychology‐centric framework and under significant structural constraints. These pressures lead to occupational therapists being forced to take on the additional role of the advocate.

Occupational therapists practising under Better Access described working with clients with complex, chronic, or comorbid mental health needs. This complexity was due in part to challenges with accessing appropriate services but was primarily compounded by macro socio‐economic factors such as unstable housing, lack of social supports, and experiences of trauma, as well as co‐occurring physical health challenges (Macintyre et al., 2018). This was an unexpected finding as it directly contrasts with the published aims of the initiative, which are to work with people with mild to moderate mental illness (Department of Health, 2025). Australia's mental health system largely prioritises very low‐intensity interventions or acute hospital‐based care and subsequently fails to meet the needs of those who fall between these two ends of the continuum (Rosenberg & Harvey, 2021; Menssink et al., 2025). This ‘missing middle’ service gap has meant that occupational therapists are working with clients whose needs exceed what the Better Access funding model allows them to provide.

As holistic, practical, and recovery‐oriented health professionals, occupational therapists are uniquely equipped to support people with these more complex presentations (D'Amico et al., 2018; Read et al., 2018; Scanlan et al., 2017). Their focus on everyday functioning, meaningful activity, and collaborative goal‐setting enables them to provide value in areas where Australia's mental health system often falls short. A recovery‐oriented approach depends on people being supported to identify and articulate their priorities and to then have access to services best suited to those priorities. This becomes difficult when system‐level restrictions limit pathways to appropriate care (Ellison et al., 2016).

Although the NDIS can fund occupational therapy for people with enduring psycho‐social disability, there are significant challenges with access, including a disconnect between the concepts of recovery and the premise of permanent disability that underpins the NDIS. Accessing the NDIS requires navigating a complex eligibility process, and even when approved, the scheme does not interface well with mainstream mental health or primary care services (Choi et al., 2025; Mellifont et al., 2023). As a result, the NDIS cannot compensate for the limitations of Better Access, leaving many people with complex mental health needs without appropriate, coordinated pathways to occupational therapy.

The full title of the initiative, *Better Access to Psychiatrists, Psychologists and General Practitioners through the Medicare Benefits Schedule*, reflects its original design focus, positioning clinical psychologists as the primary allied health providers within the scheme (Mathews, 2018). The absence of occupational therapy and social work from the title, despite both professions being eligible to deliver services under Better Access (Department of Health, 2025), continues to shape how occupational therapists are valued, recognised, and utilised within the initiative. This structural omission reinforces longstanding patterns of sidelining, where occupational therapists' contributions to mental health are overlooked or misunderstood. Such undervaluing is not unique to Better Access or to Australia; it reflects a broader international trend in which occupational therapy remains under‐recognised within mental health systems (Alotaibi et al., 2024; Henderson et al., 2015; Jesus et al., 2025; Kamat & Vajaratkar, 2020).

Due to a lack of awareness, referrers commonly default to psychology when creating mental health care plans, leading to occupational therapists receiving fewer or later referrals, if any. This has significant implications as recovery‐focussed care requires clients to access the professional whose approach best aligns with their goals and priorities (Tan et al., 2018; Tepavicharov et al., 2022). When referrals default to a single profession, clients lose access to the tailored, occupation‐focussed supports that may be more appropriate for their recovery.

Occupational therapists practising under Better Access are restricted to delivering focussed psychological strategies not aligned with the profession's core values (Davis‐McCabe et al., 2019; Holmes et al., 2023). Occupational therapists worldwide have reported that mental health services prioritise biomedical and psychological models, with limited support for long‐term or occupation‐centred care (Jesus et al., 2025). Although such issues exist across mental health occupational therapy practice (Ashby et al., 2016), they are intensified under the Better Access model, requiring occupational therapists to work within models that do not fully reflect recovery‐oriented practice.

Despite the constraints of Better Access, all participants highlighted that they could continue to integrate their occupational lens actively and intentionally (Goh et al., 2019). For example, although participants described constraints associated with delivering focussed psychological strategies, they also highlighted the ways they adapted approaches to support real‐world functioning and everyday occupations. This aligns with Beck's (1995) original framing of cognitive therapy, which emphasised applying cognitive and behavioural strategies to meaningful, real‐life activities. Recognising this alignment strengthens the case for ensuring that Better Access guidelines support occupation‐centred applications of techniques such as cognitive behaviour therapy rather than narrowly defining psychological techniques.

Session caps significantly undermine recovery‐oriented care by forcing therapy into a symptom‐focussed, time‐pressured model, a concern repeatedly raised by all health professionals working under the initiative (Mathews, 2018; Pirkis et al., 2022). A core element of recovery‐oriented practice is facilitating connection by supporting people to build and sustain relationships that enable participation, belonging, and safety (Burger et al., 2024; Isaacs, 2021). The Better Access funding model does not allow occupational therapists to collaborate with family members, carers, or other key supports without using a client's limited session allocation. This exclusion directly contradicts recovery‐oriented principles, which emphasise shared understanding, coordinated care, and relational support. These constraints are intensified by the comparatively low rebates available to occupational therapists, even though they are required to use the same focussed psychological strategies as general psychologists (Medicare Benefits Schedule, 2025a, 2025b). Additionally, many participants reported feeling obligated to bulk‐bill clients experiencing financial difficulties, further impacting the viability of their practices.

### 4.1 Enhancing the contributions of occupational therapy within Better Access

Occupational therapists practising within the first 12 months of Better Access' inception in 2006 echoed these feelings of being undervalued and not recognised, dissatisfied with remuneration, and constrained in practising under focussed psychological strategies (Hitch, 2009). Nearly 20 years on, occupational therapists are still feeling the pressure of working within a constrained, psychology‐centric system, despite Hitch's (2009) hopes of improvement. The need for advocacy work continues to create ongoing challenges for occupational therapists. Advocacy is embedded in occupational therapy theory, philosophy, and practice (Kirsh, 2015; Restall et al., 2022). However, the conflict inherent in Better Access is the need to navigate the competing demands of advocating for their clients, for the profession, and for the legitimacy of occupational therapy within a system misaligned with sustainable, recovery‐oriented practice.

A growing body of research has documented structural limitations within Better Access, and our findings align closely with these evaluations. The independent evaluation by Pirkis et al. (2022) identified that the initiative is poorly equipped to meet the needs of people with complex or chronic mental health conditions, reinforcing the ‘missing middle’ gap described by participants in this study. Consistent with our findings, the evaluation highlighted inequities in access, limited collaboration across providers, and the constraints imposed by session caps and fee‐for‐service funding. Our study extends this work by demonstrating how these system‐level limitations uniquely shape occupational therapy practice, particularly the tension between recovery‐oriented, occupation‐centred care and the psychology‐centric design of the scheme.

The research findings illustrate the need for broader workforce reform. Increasing awareness of occupational therapy's role within mental health and Better Access, and educating referrers and members of the community, would ensure that clients can access services most appropriate to their unique needs. By embedding occupational therapy explicitly within the policy language of the initiative, initially through simply the inclusion of occupational therapy within the full title of Better Access, occupational therapy is immediately given more weight and credibility as a core mental health profession within the initiative.

To further ensure occupational therapists are both valued and able to financially sustain their practice, a review of Medicare rebate structures under Better Access is essential. Aligning occupational therapy rebates with those of registered psychologists would address current inequities and support viable practice. Furthermore, the scope of practice under Better Access must be broadened. Current restrictions on focussed psychological strategies limit occupational therapists' ability to deliver the full range of their expertise. Recognising the unique skills and interventions occupational therapists provide would allow occupational therapists to practise to their full scope and provide clients access to more holistic, recovery‐oriented care.

The findings also draw attention to the Occupational Therapy Australia Mental Health Endorsement Program (Occupational Therapy Australia, 2025), which currently emphasises competency in focussed psychological strategies as required under Better Access. Although this training supports safe and evidence‐based practice within the scheme, participants' accounts suggest an opportunity for the professional body to advocate for the inclusion of occupation‐based approaches within endorsement requirements and future policy discussions. Strengthening this alignment may help ensure that occupational therapy's distinct contribution is more fully recognised within federally funded mental health models.

Finally, the findings also point to broader policy issues that extend beyond the Better Access initiative. Occupational therapists operate within a fragmented funding landscape in which Medicare, the NDIS, state‐funded community services, and private insurance each impose different eligibility rules, scopes of practice, and constraints on collaboration. These structural inconsistencies shape not only what care can be delivered but also who receives it, how recovery‐oriented principles are enacted, and where families and carers can be meaningfully involved. The study's insights highlight the need for policy reform that better aligns funding mechanisms with holistic, relational, and participation‐focussed mental health care, ensuring that occupational therapy can contribute fully to recovery‐oriented practice across the system.

### 4.2 Limitations

The use of in‐depth qualitative interviews enabled rich accounts of occupational therapists' experiences within the Better Access initiative, capturing nuances in practice, policy tensions, and the realities of delivering mental health care in constrained systems. Occupational therapists work in a wide range of mental health contexts so although participants represented diverse practice settings, some practice areas may be over‐represented or under‐represented in this dataset. The study was also unable to include GPs or other Better Access referrers, whose perspectives would have offered additional insight into referral practices. The involvement of Author 1 as a lived experience researcher strengthened the study's interpretive lens, but including other consumer voices may have deepened understanding of the lived experience of accessing occupational therapy mental health care and strengthened the relevance, interpretation, and applicability of the findings (Banner et al., 2019).

### 4.3 Implications for practice and future research

This study provides current, practical insight into an under‐researched area of occupational therapy practice, allowing the perspectives of occupational therapists working under Better Access to inform potential further research and policy development. Even without funded time for collaboration, therapists may benefit from developing streamlined communication processes with GPs and other referrers to support continuity of care within the constraints of the model. The findings also underscore the importance of professional advocacy for funding structures that recognise occupational therapy's distinct contribution to mental health care. Clinicians and professional bodies can use this evidence to argue for rebates that reflect the scope and complexity of occupational therapy practice.

Future studies should prioritise consumer perspectives to deepen understanding of how people experience occupational therapy mental health care, including barriers to access, perceptions of value, and the impact of session caps on recovery. Research involving GPs and other referrers participating in the initiative would build understanding of how occupational therapy is understood, utilised, and constrained within the broader Better Access scheme and identify opportunities for improved collaboration. There is also an opportunity to undertake policy analysis that maps how major funding mechanisms for mental health occupational therapy intersect to identify gaps, overlaps, and structural inconsistencies across schemes such as Better Access, the NDIS, state‐funded community services, and private insurance. This will provide further clarity where people fall through the system, to inform recovery‐aligned reform.

## 5 CONCLUSION

Occupational therapists are well suited to contribute meaningfully under Better Access. Their broad skill set, along with a holistic, practical, and client‐centred approach, aligns with the complex needs of the people they work with. However, the design of the initiative constrains this potential. By privileging psychology, limiting session numbers, and excluding funded time for collaboration with families, carers, and other providers, Better Access restricts the scope of occupational therapy and narrows the type of care clients can receive. These structural limitations mean that many people miss out on the purposeful, practical, and recovery‐oriented care that could strengthen their mental health and everyday participation. The existence of the ‘missing middle’ reflects deeper systemic gaps across the Australian health‐care system, where fragmented funding, inconsistent eligibility criteria, and limited continuity of care contribute to unmet needs leading to increased demand on acute services and poorer long‐term outcomes. Occupational therapists provide clear systemic value to clients, but their contribution is constrained by the current Better Access design. True recovery‐oriented care requires choice, accessibility, relational support, and interdisciplinary collaboration elements that Better Access, in its current form, does not fully enable.

## AUTHOR CONTRIBUTIONS

**Arabella Hely**: Conceptualization; investigation; formal analysis; writing—original draft; writing—review and editing. **Claire Pearce**: Conceptualization; formal analysis; writing—review and editing.

## CONFLICT OF INTEREST STATEMENT

Author Claire Pearce is an Editorial Board member for the Australian Occupational Therapy Journal.

## ETHICS APPROVAL

The ethical aspects of this study were approved by the University of Canberra Human Research Ethics Committee (Project ID: 13377).

## DECLARATION OF USE OF ARTIFICIAL INTELLIGENCE

No GenAI tools or services were used in the preparation of this manuscript other than for the Plain Language Statement as per journal guidelines. Microsoft Copilot was used to generate a plain language summary. An initial summary was inputted into Copilot with the instructions to convert to a readability score of Flesch Kincaid Level 8. The output was checked, refined, and confirmed by the authors before inclusion.

## Supporting information


**Appendix S1:** INTERVIEW GUIDE.

## Data Availability

The data that support the findings of this study are available on request from the corresponding author. The data are not publicly available due to privacy or ethical restrictions.
