# Supplementary material for: Occupational therapists' experiences in Australia's primary mental health funding scheme: Navigating complexity in a constrained system
Source: Aust Occup Ther J. 2026 Jul 1;73(4):e70104. doi: 10.1111/1440-1630.70104 (PMC13322043; doi:10.1111/1440-1630.70104)
Supplement: Supplementary file 1 — Appendix S1: INTERVIEW GUIDE. [file AOT-73-0-s001.docx]

**Supplementary File**

**APPENDIX 1: INTERVIEW GUIDE**

*This research is being conducted to explore the perspectives of occupational therapists delivering mental health care under the Better Access initiative.*

*I am seeking to understand your experiences working within the Better Access initiative, including your role, referral processes, and the opportunities or challenges you encounter when providing occupational therapy to individuals experiencing mental health concerns. Your insights will contribute to a broader understanding of occupational therapy’s contribution to primary mental health care and identify potential barriers to referral and implementation.*

| **Primary question** | **Follow up questions** |
| --- | --- |
| How would you describe your role as an occupational therapist working under Better Access? | - What types of interventions or supports do you typically provide? - What are the client groups you primarily work with? - Do you work under other funding models? - Are there limitations to what you feel you can offer in this setting? |
| Where do your referrals typically come from? | - Are they primarily from GPs? - Have you ever encountered difficulties or inconsistencies in receiving referrals under Better Access? - What factors do you think influence whether GPs refer to occupational therapy? |
| What are the main challenges or enablers you experience working under the Better Access model? | - Are there any systematic or administrative barriers that affect your work? - What supports (e.g. workplace culture, multidisciplinary teams, funding) assist in delivering occupational therapy in this context? - Have you seen any changes in referral patterns or service demand over time? - What has been your experience of obtaining Better Access accreditation? |
| In your view, what is the value or unique contribution of occupational therapy within primary mental health care? | - Are there aspects of the role that are under-recognised or misunderstood by other health professionals? - What would help strengthen occupational therapy presence or recognition under the Better Access scheme? - Do you have any recommendations to improve referral pathways or interprofessional collaboration? |
